# Extracellular vesicles in osteoarthritis: mechanisms, therapeutic potential, and diagnostic applications

**DOI:** 10.3389/fimmu.2025.1595095

**Published:** 2025-08-13

**Authors:** Chongxiao Sun, Fei Teng, Yayi Xia

**Affiliations:** ^1^ Department of Orthopaedics, Lanzhou University Second Hospital, Lanzhou, Gansu, China; ^2^ Orthopaedics Clinical Medicine Research Center of Gansu Province, Lanzhou, Gansu, China; ^3^ Intelligent Orthopedics Industry Technology Center of Gansu Province, Lanzhou, Gansu,, China

**Keywords:** osteoarthritis, extracellular vesicles, mesenchymal stem cells, stem cell-derived exosomes, therapeutic carriers, diagnostic biomarkers, anti-inflammation

## Abstract

Osteoarthritis (OA) is a chronic joint disease characterized by cartilage degradation, inflammation, and bone structural changes, leading to significant disability. Current therapeutic strategies, including traditional treatments and stem cell-based therapies, face limitations such as inability to prevent disease progression, immunogenic rejection, and tumorigenic risks. Extracellular vesicle (EVs), nanoscale membrane-bound vesicles secreted by cells, has emerged as a promising cell-free therapeutic approach due to their low immunogenicity, stability, and ability to mediate intercellular communication. This review summarizes the roles of EVs derived from various cell types, including cartilage progenitor cells (CPCs), bone marrow mesenchymal stem cells (BMSCs), synovial mesenchymal stem cells (SMSCs), adipose-derived stem cells (ADSCs), and immune cells, in OA pathogenesis and treatment. EVs exhibit multifaceted therapeutic potential, including immunomodulation, chondrocyte regeneration, and anti-inflammatory effects. Additionally, EVs serve as diagnostic biomarkers, offering non-invasive early detection of OA. Despite their promise, challenges such as scalability, targeting efficiency, and safety concerns remain. This review highlights the potential of EVs as both therapeutic agents and diagnostic tools, paving the way for innovative OA management strategies.

## 1 Introduction

Osteoarthritis (OA), as a prevalent chronic whole-joint disease, is characterized by low-grade systemic inflammation, degeneration of joint-associated tissues (such as articular cartilage), and ultimately, bone structural alterations leading to disability (1, 2). The degradation of articular cartilage is recognized as a hallmark of OA. Clinical factors such as trauma, obesity, and congenital abnormalities contribute to pathological conditions that impair the load-bearing capacity of cartilage and lead to chronic diseases such as OA (3, 4). With the aging population and rising obesity rates, the incidence of OA is increasing, imposing a substantial burden on individuals and socioeconomic systems. However, current therapeutic strategies for OA remain limited, encompassing both conventional treatments and stem cell-based therapies (5, 6). Traditional OA management includes non-surgical interventions, such as surgical procedures, and nonsteroidal anti-inflammatory drugs, including advanced-stage joint replacement (7–9). Unfortunately, these methods fail to address early disease initiation, halt cartilage degradation, or promote tissue regeneration (10). Novel treatment approaches, particularly those involving stem cell applications, encounter substantial obstacles such as immune rejection risks and potential tumor formation (11). Consequently, comprehensive insights into the causative elements and biological processes driving OA pathogenesis are crucial for formulating enhanced prevention and treatment approaches.

Extracellular vesicle (EVs) are nanoscale membrane-bound vesicles actively secreted by cells (12), capable of delivering genetic information from donor cells and mediating intercellular communication (13). EVs are produced through various biological processes, with their formation primarily stemming from the plasma membrane, which contributes to their minimal immunogenic properties (14, 15). These vesicles not only inherit most of the functional attributes of their parental cells but also circumvent several associated challenges, such as immune-compatibility, stability, heterogeneity, and the maintenance of stemness (16). Given the substantial limitations and risks associated with both conventional and stem cell-based therapies, EVs have garnered increasing attention as a cell-free therapeutic strategy for OA (1). Accumulating evidence suggests that EVs play a crucial and multifaceted role in OA pathogenesis, diagnosis, and treatment. This review provides a comprehensive overview of the role of EVs from various sources in OA and their potential applications in OA therapy.

## 2 The roles of different EVs in OA

EVs derived from various cell types exert distinct roles in the treatment of OA. In normal physiological processes, OA repair and tissue regeneration encompass multiple mechanisms, including immune regulation, pain management, reduction of chondrocyte aging and metabolic imbalance, as well as stimulation of cartilage cell renewal (17, 18). In addition to their direct effects on chondrocytes, EVs interact extensively with synovial fibroblasts, the synovium, and subchondral bone. By modulating synovial fibroblast activity, EVs can reduce production of pro-inflammatory cytokines and inhibit the recruitment of immune cells (19). EVs also influence subchondral bone remodeling by regulating osteoclastogenesis and osteoblast activities via pathways such as RANKL-RANK-OPG (20). Furthermore, EV cargo containing proteolytic enzymes or their inhibitors can reshape the local extracellular matrix (ECM) environment, balancing matrix synthesis and degradation (21). Through this multidimensional interplay, EVs help restore joint homeostasis, thereby exerting regenerative and anti-inflammatory actions across multiple tissue interfaces in OA. EVs derived from various cellular origins demonstrate distinct biological characteristics. The following analysis focuses on the functional contributions of EVs produced by different cell types in OA.

### 2.1 Synovial mesenchymal stem cells derived EVs

Synovial mesenchymal stem cells have been demonstrated to attenuate OA progression. With superior proliferation and chondrogenic potential, SMSCs facilitate cartilage repair by accelerating chondrocyte proliferation (22). SMSCs promote cartilage repair by accelerating chondrocyte proliferation and differentiation, and their chondrogenic potential is attributed to several key factors (23). Furthermore, SMSCs exhibit tissue specificity for cartilage regeneration, underscoring their potential applications in cartilage repair (24). SMSCs express high levels of chondrogenic markers, including Sox9, collagen type II (COL2A1), and aggrecan, which are essential for cartilage formation (25). Mechanistically, SMSCs exhibit enhanced chondrogenic differentiation through the activation of the Wnt signaling pathway, which mediate the activation of the Yes-associated protein (YAP) pathway in chondrocytes (26–29). These signaling cascades stimulate chondrocyte proliferation and matrix synthesis, promoting cartilage regeneration.

A study revealed that extracellular vesicles secreted by lipopolysaccharide (LPS)-preconditioned SMSCs (LPS-pre-EVs) promote chondrocyte proliferation and migration while inhibiting apoptosis. This effect is primarily mediated by suppressing IL-1β-induced aggrecan and COL2A1 degradation and reducing ADAMTS5 expression (30). In a murine OA model, LPS-preconditioned EVs delayed early OA progression and prevented OA-induced knee cartilage damage *in vivo*. Given the reduced expression of miR-129-5p and the upregulation of HMGB1 in OA patients and IL-1β-induced chondrocytes, which stimulate inflammatory and apoptotic responses (31, 32). Exosomes are a subtype of EVs with a diameter typically ranging from 30 to 150 nm (33). Another study has demonstrated that SMSC-derived exosomes (SMSC-Exo) with high miR-129-5p expression significantly alleviated chondrocyte inflammation and apoptosis, whereas low miR-129-5p expression exacerbated IL-1β-mediated chondrocyte inflammation and apoptosis. This mechanism is primarily mediated through the suppression of HMGB1 release by miR-129-5p in SMSC-Exo, thereby inhibiting IL-1β-induced OA pathogenesis (34). Furthermore, SMSC-derived extracellular vesicles were found to contain abundant Wnt5a and Wnt5b, which activate YAP through non-canonical Wnt signaling pathways (35). Collectively, these findings establish SMSC-EVs as a promising acellular therapeutic strategy for OA, capable of simultaneously promoting chondrocyte differentiation, migration, and proliferation while inhibiting apoptotic processes.

### 2.2 EVs derived from bone marrow mesenchymal stem cells

BMSCs are a population of multipotent stem cells with adipogenic, osteogenic, and chondrogenic potential (36). They exhibit regenerative capacity and immunomodulatory functions and have been utilized in treating inflammatory and degenerative diseases such as OA, rheumatoid arthritis, and bone defects (36, 37). BMSC-EVs exert their anti-inflammatory and regenerative effects by modulating pathways such as NF-κB, which governs the release of pro-inflammatory cytokines (38). Besides, BMSC-EVs significantly mitigate IL-1β-induced suppression of chondrocyte proliferation and motility *in vitro* (39). Furthermore, EV-based treatment effectively counteracts the IL-1β-mediated upregulation of MMP13 and a disintegrin and metalloproteinase with thrombospondin motifs (ADAMTS), while preventing the downregulation of collagen type II and aggrecan (35). Among EVs explored for OA therapy, BMSC-derived EVs (BMSC-EVs) are the most extensively studied. A recent study demonstrated that BMSC-EVs induce cartilage reconstruction in OA via the autotaxin-YAP signaling axis. Specifically, sEVs-autotaxin promotes cartilage repair and upregulates key Hippo pathway regulators (40). BMSC-EVs also facilitate cartilage defect repair by promoting cell proliferation and infiltration and modulating cellular functions through various miRNAs (41, 42). When co-cultured with OA chondrocytes, BMSC-EVs exhibit high expression of cyclooxygenase-2 (COX-2) and pro-inflammatory interleukins while inhibiting tumor necrosis factor-α (TNF-α)-induced collagen degradation and promoting ACAN and collagen II synthesis (43). Collectively, BMSC-EVs display substantial regenerative and immunoregulatory properties in OA cartilage, making them an ideal candidate for OA treatment.

### 2.3 EVs derived from cartilage progenitor cells

CPCs possess high self-renewal capacity and chondrogenic potential (44). Cellular populations with mesenchymal stem cell (MSC)-like properties, particularly CPCs and cartilage-derived stem cells, play pivotal roles in both cartilage formation and its regulatory processes due to their oligopotent differentiation capacity (45). Serving as cartilage progenitor cells, CPCs play a pivotal role in maintaining cartilage homeostasis. Moreover, CPCs may significantly impact OA progression by mitigating chondrocyte proliferation and cartilage formation (46). Changes in their spatial organization during OA development indicate their potential role in mediating cellular interactions among articular cartilage, subchondral bone, and adjacent joint components (44). An *in vitro* study demonstrated that CPCs effectively reduce inflammatory cytokines (IL-6, MCP-1, and IL-1β) and matrix metalloproteinases (MMPs) while significantly upregulating collagen II expression in IL-1β-induced human chondrocytes (47). Additionally, CPCs exhibit lower expression of the transcription factor RUNX2, which is essential for chondrocyte terminal differentiation and calcified bone formation (48). Consequently, CPCs resist hypertrophy and continuously produce hyaline-like cartilage.

A recent study applied CPC-derived EVs (CPC-EVs) in OA for the first time, comparing their therapeutic effects with MRL-EVs and normal murine EVs. This study also investigated the impact of CPC-EVs and MRL-EVs on chondrocyte proliferation and migration *in vitro* and *in vivo* (49). Unlike BMSC-EVs, which primarily act through the autotaxin-YAP signaling axis and Hippo pathway to induce cartilage reconstruction (40), CPC-EVs exhibit a more direct ECM-modulatory role, enhancing type II collagen synthesis in inner meniscal fibrochondrocytes and promoting cellular regeneration without significant involvement of hypertrophic pathways (50, 51). Recent studies have demonstrated that EVs derived from CPC-EVs exhibit a preferential localization to cartilage tissue following intra-articular injection (52). These EVs have been shown to promote matrix anabolism and inhibit inflammatory responses, at least partially by blocking STAT3 activation, thereby enhancing cartilage repair mechanisms. Intra-articular secreted EVs from CPCs may impede OA progression, paving the way for novel therapeutic strategies.

### 2.4 EVs derived from adipose mesenchymal stem cells

ADSCs possess regenerative capabilities akin to BMSCs. However, ADSCs have gained increasing attention due to the ease of adipose tissue harvesting and the relatively simple cell isolation process, yielding approximately 500 times more stem cells than bone marrow (53). Several studies have investigated the therapeutic potential of ADSCs in OA (54, 55). Early studies demonstrated that intra-articular injection of ADSCs confers anti-inflammatory, antioxidative, and chondroprotective effects (56, 57). Recent findings indicate that ADSC-derived EVs (ADSC-EVs) primarily function by preserving chondrocytes and suppressing inflammation. ADSC-EVs promote human OA chondrocyte proliferation and migration while modulating catabolic and anabolic factors and effectively preventing macrophage infiltration into synovial tissue (58). Inflammatory factors are pivotal in inflammatory diseases progression (59, 60). ADSC-derived EVs regulate gene expression and protein secretion in chondrocytes and synoviocytes, effectively counteracting IL-1β-induced inflammatory responses and mitigating NF-κB pathway-mediated inflammatory and catabolic environments, offering a promising strategy for OA treatment (61). Additionally, miRNAs present in ADSC-EVs have been implicated in OA pathogenesis (62). Hence, ADSC-EVs should be considered a potential therapeutic approach for OA.

### 2.5 EVs derived from other cell types

**Figure 1:**
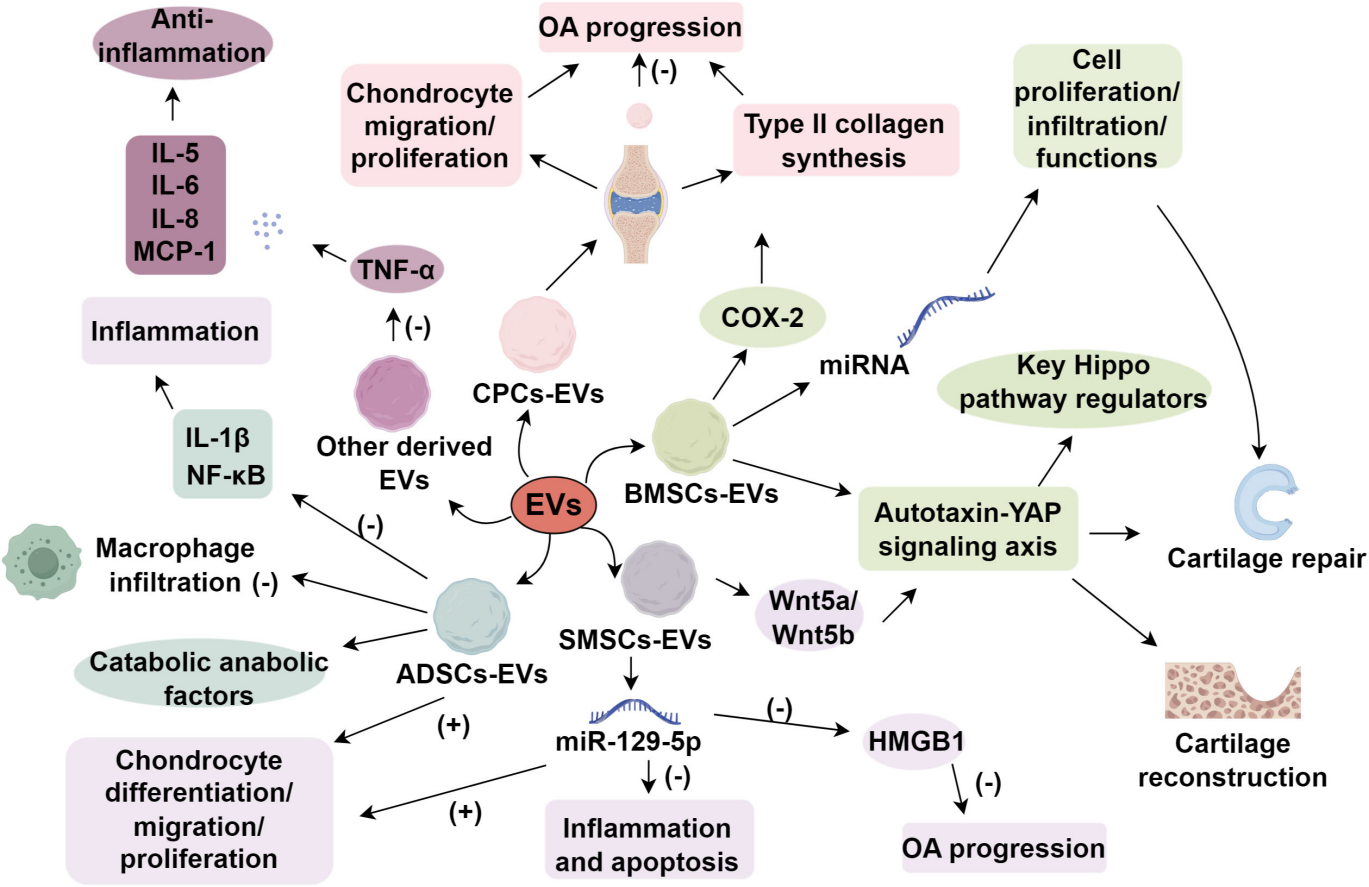
Mechanisms of extracellular vesicles in osteoarthritis.

Comprehensive investigations into EVs from diverse cellular origins are essential for developing robust therapeutic strategies and advancing our understanding of OA pathogenesis. Immune cells, such as neutrophils and macrophages, influence the inflammatory milieu and chondrocyte senescence and metabolism in OA. Compared to stem cell-derived EVs, immune cell-derived EVs typically exhibit simpler functionality, potentially minimizing adverse effects (63). Neutrophil-derived EVs have been shown to be internalized by fibroblast-like synoviocytes in OA patients, thereby downregulating TNF-α-induced inflammatory cytokines such as IL-5, IL-6, IL-8, and MCP-1, exerting an anti-inflammatory effect (64). However, not all immune cell-derived EVs exhibit protective roles (65). Recent studies have revealed that EVs secreted by pro-inflammatory macrophages in the osteoarthritic synovium can carry potent inflammatory cargo, such as IL-1β, contributing to local joint inflammation and cartilage degradation (66). These IL-1β^+^ macrophage-derived EVs can enhance the activation of fibroblast-like synoviocytes and upregulate MMPs and other catabolic mediators, thereby exacerbating synovial inflammation and OA progression (67, 68). This highlights the dual, context-dependent nature of immune-derived EVs, which can either attenuate or aggravate OA pathology depending on their cellular origin and microenvironmental stimuli. Moreover, antler stem cell-derived exosomes (ASC-Exos) restore heterochromatin stability and rejuvenate senescent MSCs, offering a novel OA treatment strategy (69). While EVs from various sources present diverse therapeutic potentials for OA, rigorous preclinical studies, including long-term efficacy assessments and safety evaluations, are imperative before clinical translation (**Figure 1**).

## 3 Application of EVs in osteoarthritis treatment

### 3.1 Potential of EVs as therapeutic carriers

Natural EVs are widely investigated for osteoarthritis therapy due to their accessibility and effectiveness in promoting cartilage regeneration and joint repair, yet challenges such as poor targeting, scalability, and safety hinder clinical translation, driving research into engineered EVs as optimized drug delivery systems (70, 71). Currently, engineering strategies are being developed to improve EV-mediated drug delivery, targeting precision, and therapeutic efficacy (72). Methods for loading exogenous molecules into EVs can be broadly categorized into two approaches (73, 74). The first approach involves modifying donor cells using biochemical factors or mechanical factors. The second strategy entails direct EV modifications, including exogenous cargo loading and membrane engineering.

Moreover, numerous studies have demonstrated that MSC-derived exosomes contain bioactive molecules, such as miR-8485, miR-95a-5p, miR-320c, miR-150-5p, and miR-26a-5p, which regulate pathways such as Wnt/β-catenin and RANKL-RANK-TRAF6 by targeting proteins including MMPs, histone deacetylase 2 (HDAC2), and COX-2. These regulatory effects help mitigate inflammation, maintain ECM stability, reduce OA chondrocyte apoptosis, and promote chondrocyte proliferation and migration, thereby alleviating joint damage (75–81). Furthermore, immune responses within joint cartilage play a crucial role in OA progression. Evidence suggests that MSC-derived exosomes exert immunomodulatory effects by suppressing T lymphocytes while activating B lymphocytes, highlighting their potential in immune regulation and making this a promising avenue for future research (82). Notwithstanding their generally low immunogenicity, EVs may still pose immunological risks under certain conditions. If EVs carry unexpected alloantigens pathogen-derived components, or pro-inflammatory molecules (83, 84), they could inadvertently trigger immune activation, particularly in individuals with underlying autoimmune disorders or compromised immune tolerance. Hence, stringent purification, characterization, and screening protocols are vital to minimizing adverse immunologic reactions in EV-based therapies.

### 3.2 Application of EVs as diagnostic biomarkers

Currently, OA diagnosis relies on clinical symptoms and physical examinations, with imaging techniques such as radiography used as supplementary tools when necessary (85). However, early-stage OA is often asymptomatic, and treatment becomes increasingly challenging as the disease progresses. Therefore, there is an urgent need for biomarkers to facilitate early diagnosis (86). Blood sampling provides a minimally invasive approach, and given that EVs remain stable in body fluids, they hold promise as screening tools for early-stage OA. EV-based diagnostics could enable non-invasive assessment of joint burden and potential risk factors before imaging-confirmed OA manifestations. Additionally, early biomarker-based diagnosis could offer insights into cellular and molecular alterations, facilitating timely and targeted interventions. EVs are promising novel biomarkers due to their ability to encapsulate donor cell-derived molecular signatures while maintaining strong stability within the circulatory system (86). Compared with existing OA biomarkers such as cartilage oligomeric matrix protein (COMP) (87), C-reactive protein (CRP) (88), and certain pro-inflammatory cytokines (89), EV-derived markers may offer higher sensitivity and specificity by virtue of their cell-type-specific cargo (90). Their encapsulated structure not only prolongs half-life in the circulation but also preserves the integrity of proteins and nucleic acids that can be degraded in free form. Additionally, EV-based biomarkers may allow for earlier detection of OA by capturing dynamic changes in gene expression and protein composition within the joint environment, thus providing a more refined molecular fingerprint of disease progression (91). Studies have demonstrated that plasma-derived exosomal miR-193b-3p expression is significantly reduced in OA patients compared to healthy individuals, mirroring findings from degenerative cartilage samples (92). Furthermore, previous research has indicated an inverse correlation between serum miR-142-5p expression and inflammatory responses, suggesting that plasma exosomal miR-142-5p represents a potential biomarker for OA (93). However, given that these miRNAs also exhibit differential expression patterns in rheumatoid arthritis (94), the development of a multi-miRNA panel would significantly improve the accuracy of clinical differential diagnosis between these two arthritic conditions.

The advancement of EV-derived biomarker systems, incorporating diverse molecular components such as genetic material, protein complexes, lipid structures, and carbohydrate molecules, has demonstrated significant potential across multiple medical domains, especially in cancer research, metabolic syndrome investigations, and neurological disorder studies (95–97). A notable example is the ExoDx™ Lung test, which was the first EV-based biomarker to undergo clinical trials in 2016 for detecting EML4-ALK mutations in lung cancer diagnosis (98). This milestone underscored the superior diagnostic potential of EVs. In the context of OA, synovial EVs have been identified as markers for differentiating disease stages, further expanding their diagnostic value (99). Initial studies on EVs as OA biomarkers emerged from mechanistic investigations, revealing differential miRNA and other nucleic acid expressions between healthy individuals and OA patients (100). Subsequently, EVs were found to facilitate OA subtype differentiation, introducing a novel paradigm for clinical diagnosis (101). Furthermore, the isolation of exosomes derived from biological fluids such as blood and urine, followed by the characterization of their cargo, has emerged as a promising approach for identifying biochemical biomarkers to predict cartilage degeneration and assess the progression of joint-related diseases. Recent studies have extracted synovial fluid-derived exosomes from patients with OA and performed RNA sequencing analysis on their miRNA content, revealing a significant upregulation of miR-210-5p (102). Further investigations have demonstrated that miR-210 is markedly upregulated in synovial fluid samples from both early- and late-stage OA patients and is positively correlated with VEGF levels. These findings suggest that the upregulation of miR-210 in synovial fluid may occur at the early stages of OA, highlighting its potential as a non-invasive and early diagnostic biomarker for identifying individuals at risk of developing OA and enabling rapid disease detection (103). EVs have already been employed in clinical research for disease diagnosis, progression monitoring, and prognostic evaluation. These findings underscore the robust potential of EVs as biomarkers, and it is anticipated that EV-based OA biomarker applications will be realized in clinical practice in the near future (**Table 1**).

**Table 1:** Potential and challenges of EVs in osteoarthritis treatment and diagnosis.

EV Source	Target	Mechanism	Benefits	Limitations
MSC-Derived EVs	miR-8485, miR-92a-3p, miR-320c, miR-140-5p, miR-26a-5p	Regulate Wnt/β-catenin, RANKL-RANK-TRAF6 pathways, and target MMPs, HDAC2, COX-2	Alleviate inflammation; Maintain ECM stability; Promote chondrocyte proliferation and migration; Mitigate joint damage	Limited targeting specificity; Difficulties in large-scale production and purification; Potential safety risks due to heterogeneity
Engineered EVs	Loaded with exogenous drugs/genes through biochemical or/mechanical factors/direct EV modification (e.g., electroporation, membrane engineering)	Surface modification to enhance tissue/cell targeting; Increased loading efficiency of exogenous molecules	Improved specificity for OA treatment and reduced immunogenicity; Enhanced drug accumulation and tissue repair at the target site	1Need for optimized loading efficiency and drug stability; Long-term safety and immunological profiles remain to be fully assessed
Plasma-Derived EVs	miR-193b-3p	Expression inversely correlated with inflammatory responses	Potential early screening marker for OA; Reflects molecular changes related to cartilage degeneration	Larger clinical studies needed to confirm specificity and sensitivity; Lack of standardized detection methods
Synovial Fluid-Derived EVs	miR-210-5p	Positively correlated with vascular endothelial growth factor (VEGF) levels	Early diagnosis and staging of OA; Evaluation of synovial environment and microvascular formation	Synovial fluid collection is more invasive; Larger-scale cohort studies required for further validation;
Naturally Derived EVs (General)	Endogenous proteins, peptides, miRNAs, etc.	Reduced immune recognition through adhesion proteins and ligands on EV surface Extended circulation time	Enhanced biocompatibility and stability; Broad potential in pathological and diagnostic applications	No universal standard for large-scale production and purification; Heterogeneity may lead to variable efficacy

## 4 Conclusion

OA remains a significant global health challenge, with current therapeutic strategies offering limited efficacy in halting disease progression or promoting cartilage regeneration. EVs have emerged as a promising alternative, offering a cell-free approach to OA treatment with advantages such as low immunogenicity, stability, and the ability to mediate intercellular communication. EVs derived from various cell types, including CPCs, BMSCs, SMSCs, ADSCs, and immune cells, demonstrate diverse therapeutic potentials, including immunomodulation, chondrocyte regeneration, and anti-inflammatory effects.

Furthermore, EVs hold significant promise as diagnostic biomarkers, enabling early detection and monitoring of OA progression through non-invasive methods. However, challenges such as scalability, targeted delivery, and safety concerns must be addressed before clinical translation. Future research should focus on optimizing EV-based therapies, improving their targeting efficiency, and conducting rigorous preclinical and clinical trials to ensure their efficacy and safety. Overall, EVs represent a transformative approach to OA management, offering hope for more effective prevention, diagnosis, and treatment of OA.
